# The effect of women, infant, and children (WIC) services on birth weight before and during the 2007–2009 great recession in Washington state and Florida: a pooled cross-sectional time series analysis

**DOI:** 10.1186/s12884-020-02937-5

**Published:** 2020-04-28

**Authors:** Erin L. Blakeney, Jerald R. Herting, Brenda Kaye Zierler, Betty Bekemeier

**Affiliations:** 1 Department of Behavioral Nursing and Health Informatics, School of Nursing, University of Washington, Box # 357266, Seattle, WA 98195 USA; 2 Department of Sociology, University of Washington, Box 353340, Seattle, WA 98195 USA; 3 Department of Child, Family, and Population Health Nursing, School of Nursing, University of Washington, Box # 357263, Seattle, WA 98195 USA

**Keywords:** WIC, Birth Weight., Great Recession., Disparities.

## Abstract

**Background:**

The Special Supplemental Nutrition Program for Women, Infants, and Children (WIC) has been shown to have positive effects in promoting healthy birth outcomes in the United States. We explored whether such effects held prior to and during the most recent Great Recession to improve birth outcomes and reduce differences among key socio-demographic groups.

**Methods:**

We used a pooled cross-sectional time series design to study pregnant women and their infants with birth certificate data. We included Medicaid and uninsured births from Washington State and Florida (*n* = 226,835) before (01/2005–03/2007) and during (12/2007–06/2009) the Great Recession. Interactions between WIC enrollment and key socio-demographic groupings were analyzed for binary and continuous birth weight outcomes.

**Results:**

Our study found beneficial WIC interaction effects on birth weight. For race, prenatal care, and maternal age we found significantly better birth weight outcomes in the presence of WIC compared to those without WIC. For example, being Black with WIC was associated with an increase in infant birth weight of 53.5 g (baseline) (95% CI = 32.4, 74.5) and 58.0 g (recession) (95% CI = 27.8, 88.3). For most groups this beneficial relationship was stable over time.

**Conclusions:**

This paper supports previous research linking maternal utilization of WIC services during pregnancy to improved birth weight (both reducing LBW and increasing infant birth weight in grams) among some high-disadvantage groups. WIC appears to have been beneficial at decreasing disparity gaps in infant birth weight among the very young, Black, and late/no prenatal care enrollees in this high-need population, both before and during the Great Recession. Gaps are still present among other social and demographic characteristic groups (e.g., for unmarried mothers) for whom we did not find WIC to be associated with any detectable value in promoting better birth weight outcomes. Future research needs to examine how WIC (and/or other maternal and child health programs) could be made to work better and reach farther to address persistent disparities in birth weight outcomes. Additionally, in preparation for future economic downturns it will be important to determine how to preserve and, if possible, expand WIC services during times of increased need.

**Trial registration:**

Not applicable, this article reports only on secondary retrospective data (no health interventions with human participants were carried out).

## Background

The Special Supplemental Nutrition Program for Women, Infants, and Children (WIC) is a government-funded nutritional supplementation and education program that has generally been shown to have positive effects in promoting healthy birth outcomes in the United States [1, 2]. WIC started in 1972 in response to concerns about malnutrition among pregnant women and its impacts on their children [1–4]. Today, WIC services provide nutritional assessment, health education, food supplementation (vouchers for specific foods) and referrals for low-income pregnant and postpartum women, infants, and children up to age 5 years [1]. Food vouchers are valued at approximately $50/month for pregnant women [5]. To be eligible for WIC services, applicants must have income at or below 185% of the United States (U.S.) Poverty Income Guidelines or be enrolled in Temporary Aid for Needy Families (TANF), Supplemental Nutrition Assistance Program (SNAP), or Medicaid. Applicants are screened medically (e.g., for anemia, underweight, smoking) and for risk of nutritional deficits (e.g., low dietary consumption of protein or iron) [5]. During the Great Recession, concerns about maternal malnutrition and its impacts on birth outcomes were heightened.

The U.S. Department of Agriculture renews WIC services through annual discretionary funding by the U.S. Senate and House Appropriations Committee [6]. Funding is disbursed to states who then distribute funding to WIC provider agencies such as private nonprofits and local health departments that generally serve multi-county, county, or city local health jurisdictions. Staff in these agencies engage with individual WIC enrollees, recruit and review WIC applicant eligibility, distribute food vouchers, and make individual as well as group-based health education available to WIC enrollees. Enrollees are encouraged, but not required, to participate in group health education. Findings of numerous studies suggest that the WIC program is effective and helps to (a) reduce premature births; (b) reduce low birth weight (LBW) and very LBW babies; (c) reduce fetal and infant deaths; (d) reduce incidence of low-iron anemia; (e) increase access to prenatal care earlier in pregnancy; (f) increase pregnant women’s consumption of key nutrients such as iron, protein, calcium, and vitamins A and C; (g) increase immunization rates; (h) improve diet quality; and (i) increase regular access to health care [3, 4, 7–14].

A robust body of research exists documenting socio-demographic disparities in maternal and child health outcomes in the U.S. [15, 16]. One goal of programs like WIC is to address and reduce these disparities, and findings suggest that WIC participation can improve outcomes and narrow existing disparity gaps [3, 4, 7–9, 17]. Given the varied baselines from which different groups participate in the WIC program, previous research has also documented differential WIC effects among subpopulations. For example, Bitler and Currie (2005) and Kowaleski-Jones and Duncan (2002) found that mothers who participate in WIC are more likely to have babies with a healthy birth weight and to breastfeed their infants—with more pronounced effects among mothers with greater disadvantage (e.g., who received other forms of public assistance) [7, 8]. Other researchers have reported more pronounced WIC effects among Black mothers compared to White mothers. For example, Khanani et al. (2010) identified differential WIC effects by race for both infant mortality and preterm birth, with infants of Black women who enrolled in WIC being much less likely to die than the infants of Black women who did not enroll in WIC [9]. Further, WIC utilization in this population was associated with decreased Black/White disparities in infant mortality and WIC participants were less likely to have extreme preterm (between 20 and < 34 weeks’ gestation) deliveries [9].

WIC enrollment increased during the Great Recession of 2007–2009 and the WIC program received additional funding at the federal level through the American Recovery and Reinvestment Act [18–20]. Prior to the Great Recession, WIC served approximately 50% of infants born in the U.S. [1]. During the Great Recession, WIC enrollment increased by about 5%, despite decreases in birth rate [20]. However, WIC enrollment did not increase as much as some other federally funded programs like unemployment insurance, SNAP, or Medicaid coverage [6, 15, 21]. Further, the WIC program only used $38,000,000 of the $400,000,000 in additional program funding allocated to WIC services as part of a federal stimulus package [21]. Less than estimated caseload growth and decreased food costs during the Great Recession partially explain the under-utilization of additional WIC funding [19, 21]. Widespread reports of local health departments cutting maternal and child health-related programs (reducing hours, laying off or not hiring staff, etc.) due to budget cuts may also have contributed to reduced referrals and participant access to WIC program resources, despite federal level increases in funding [22, 23].

These changes in resource allocation and utilization, combined with previous research indicating differential WIC effects among race/ethnicity and other groups defined by social and demographic characteristics, suggest that associations between birth weight and maternal WIC enrollment may have changed during the Great Recession. In this paper we test the hypothesis that positive WIC effects on birth weight and birth weight disparities among key socio-demographic groups held just prior to (01/2005–03/2007) and during the Great Recession (12/2007–06/2009).

## Methods

Using secondary data and a pooled time series cross-sectional design, we tested whether maternal WIC enrollment had a differential association on birth weight by multiple social and demographic groups during the Great Recession. To do this we analyzed the interaction between maternal WIC enrollment with race/ethnicity and other groups defined by social and demographic factors, such as education level, on birth weight, within regression models representing pre- and intra-Recession periods. The first period (pre-Recession/baseline) was from January 2005–March 2007. The second period encompassed the official Recession dates (December 2007–June 2009) [24]. Institutional Review Board approval for this work was obtained from the University of Washington as well as the State Departments of Health in Florida and Washington State.

### Study population

De-identified, individual-level birth certificate data were obtained from the state Departments of Health in Washington and Florida with relevant human subjects approvals from both Institutional Review Boards to carry out these analyses. Washington and Florida were chosen as study states based on the availability of uniquely comparable, longitudinal public health budget data and previous studies indicating relationships between public health expenditures and beneficial maternal and child health outcomes [16, 17, 25, 26]. The study population included first-time mothers of singletons in Washington and Florida who were uninsured or for whom Medicaid paid for their births. Limiting the study population to uninsured and Medicaid births allowed us to closely approximate a WIC-eligible population, as maternal income is not available in birth certificate data. We further restricted inclusion to records that included complete WIC enrollment data (birth certificate indicated whether or not the mother was WIC-enrolled), birth weight, and county of residence information (to allow for linking with county/local health jurisdiction data) (*n* = 226,835).

### Measures

We considered two outcome measures of birth weight—a binary LBW measure (yes/no) and a continuous measure of birth weight in grams. LBW was defined as a birth weight of less than 2500 g. Birth weight was selected as the outcome measure for this study because other studies show that WIC services have an impact on birth weight and constitute one of the more reliable pieces of birth certificate data [1, 7, 8, 14, 27]. Both binary LBW and continuous birth weight in grams were included to allow for analysis of whether WIC was associated with having fewer LBW infants (a clinical and public health benchmark goal), as well as to assess overall relationships between WIC and birth weight. We limited analysis to infants with birth weight between 350 and 8000 g to ensure inclusion of infants most likely to be considered viable across jurisdictions, thus improving consistency in registration of births (as there is variation documented in the literature across jurisdictions in registration practices of births considered non-viable) [28]. Disparities were defined as differences in birth weight if seen to a greater or lesser extent between populations. Covariates were selected based on a conceptual framework reflecting multi-faceted social determinants of health, as well as on previous research that linked them to maternal and child health outcomes [16, 17, 29–31]. Individual, community, local health department expenditure measures, and state dummy variables (a categorical variable representing each state) were included (See Table 1 for a complete list). For all individual level variables included in this analysis missing-ness was less than 1.0%

**Table 1:** Covariates included in WIC and birth weight analyses, Washington State and Florida, 2005–2009.

Covariate Level	Covariate Name/Description
Individual	• Race/ethnicity: non-Hispanic White, Hispanic White, non-Hispanic Black, Asian, Other^a^ • Maternal age • Marital status (married/unmarried) • Mother foreign-born (Yes/No) • Maternal education (less than high school; high school diploma or GED; some college; not assessed (age < 20 years) • WIC (maternal WIC enrollment) (Yes/No) • Maternal insurance status (e.g., Medicaid or private insurance). • Late/No prenatal care (0-mother entered prenatal care during first trimester; 1-mother entered prenatal care after the first trimester or not at all)
Community^b^ (at the local health jurisdiction level unless otherwise indicated)	• Core Based Statistical Area (metropolitan, micropolitan, or rural) • Community poverty (binary variable 1 = local health jurisdictions with the highest percentage (top 1/3) of residents age 0–17 years in poverty by state; and binary variable 2 = lower 2/3 of residents in poverty (non-poor local health jurisdictions)^c^ • Percent of voters voting Republican (vs. Democrat or Independent) in the 2004 and 2008 presidential elections^d^ • Gini coefficient (2000 census: measure of income distribution/inequality (0–1), larger number > inequality)^e^ • Per Capita General and Family Practitioner MDs/local health jurisdictions (for years 2005, 2008, 2010)^f^ • Per capita local health jurisdiction unemployment rate^g^
Expenditure^h^	• Total local health department expenditures • WIC expenditures • Family planning expenditures • MICA services expenditures • Maternal and child health—combined expenditures^i^
State	• State-level dummy variables were created for WA and FL to capture any state-level differences

Abbreviations: *FL* State of Florida; *GED* General Education Diploma; *MD* Medical doctor; *MICA* Maternal/infant/ child/adolescent: *WA* State of Washington: *WIC* Special Supplemental Nutrition Program for Women, Infants, and Children

^a^ Race/ethnicity groups were defined using data from two separate variables (maternal race and maternal ethnicity) to create a 5-category combined race/ethnicity variable

^b^ Community level covariates were selected based on previous research or for which social determinants of health theories suggest a plausible association to maternal and child health outcomes in the context of the Great Recession; source: references [16, 17, 26, 27, 32–36]

^c^ Source: references [25, 26, 30, 32]

^d^ The voting patterns measure was intended to act as a proxy for differences in political orientation at the community level as previous research has identified Republican voters as less likely to perceive that there are people in the United States who encounter access to care issues as well as less likely to support public health reform; references [33–35]

^e^ Source: reference [36]

^f^ Source: reference [32]

^g^ Individual unemployment data were not available

^h^ Local health department-specific per capita expenditure data were included in the preliminary model as the Great Recession yielded widespread reports of budget cuts to local health departments; source: references [25, 26]. Per capita rates were calculated using total local health jurisdiction population as a denominator. Differences in fiscal years between WA and FL were reconciled by assigning FL’s fiscal year to the earlier year (e.g., FL fiscal year 2005–2006 associated with WA fiscal year 2005)

^i^ MICA represents a composite of similar budget categories for WA and FL that includes comparable intervention activities across both states—e.g., home visiting, prenatal health programs; source: references [25, 26]

### Analysis

Linear regression models were specified for both time periods (pre- and intra-Recession) as well as for each birth weight outcome (a linear probability model for the binary LBW and continuous regression model for birth weight in grams). Models were estimated using STATA 12.0 [37]. In all analyses, robust standard errors were used to account for heteroscedasticity and clustering across local health jurisdictions. WIC enrollment interactions with conceptually relevant individual level covariates were also introduced in the models and included the following factors: maternal race/ethnicity, maternal age, marital status, maternal birth place, education, timing of entry to prenatal care, payer (Medicaid vs. uninsured), and population density of local health department of residence as measured by Core Based Statistical Area (metropolitan, micropolitan, rural) [16, 17]. Interactions are typically carried out within regression models to determine whether the association of variables with an outcome (e.g., LBW, birth weight in grams) varies depending on the value of another variable or variables [38, 39]. In this case, we tested whether the association of social and demographic characteristics and birth weight changed when a mother was enrolled in WIC (or not). Throughout the results, the coefficients presented are the change in the probability that the dependent variable (i.e. that an infant would be born at a LBW) equals one for a one unit change in the independent variable (i.e. whether or not a pregnant woman was enrolled in WIC), holding everything else constant. These coefficients can be interpreted as an expected probability or expected percentage (i.e., 0.084 = 8.4%).

## Results

Table 2 summarizes the birth outcomes and key social/demographic characteristics of the study population by WIC or no WIC enrollment before and during the Great Recession. The majority of the population was enrolled in WIC, which is expected in a population of WIC-eligible pregnant women who were uninsured (self-pay) or insured through Medicaid. Similar to the nationally reported increase in WIC enrollment during the Great Recession, WIC enrollment in our study population increased by approximately 5% (from 73.8% during baseline to 78.8% during the Great Recession) [20]. Fewer of those enrolled in WIC were married and or foreign-born than non-WIC participants. Slightly fewer pregnant women enrolled in WIC numbers of entered prenatal care late (or not at all) or gave birth to LBW infants compared to non-WIC participants. Demographic and social/economic characteristics did not shift dramatically between baseline and the Great Recession periods for any group.

**Table 2:** Study population with/without WIC during baseline and Great Recession periods (n / % unless otherwise indicated)

	Baseline No WIC	Baseline WIC	Recession No WIC	Recession WIC
**Birth Weight**				
Birth weight in grams SD= n=	3179.7 (620.6) 34,485	3217 (562.2) 97,033	3199.2 (616.5) 20,236	3210.5 (561.4) 75,081
Low birth weight (< 2500 g)	3285 (9.5%)	7886 (8.1%)	1895 (9.4%)	6128 (8.1%)
**Mother’s Age**				
Mother’s age, years (mean) SD= n=	24.0 (6.0) 34,478	22.1 (5.3) 97,024	24.5 (5.9) 20,234	22.2 (5.2) 75,077
Teenage births (< 19 years) as component group of total study population	8279 (24.0%)	34,810 (35.9%)	4315 (21.3%)	26,041 (34.7%)
Teenage births (age ≤ 14 years) as component group of total study population	155 (4.5%)	653 (6.7%)	68 (3.4%)	424 (5.6%)
**Mother’s Race/Ethnicity**				
Non-Hispanic White	19,319 (56.9%)	43,515 (45.3%)	11,600 (58.4%)	33,550 (45.2%)
Hispanic White^a^	5116 (15.1%)	20,635 (21.5%)	2502 (12.6%)	14,459 (19.5%)
Non-Hispanic Black	5457 (16.1%)	22,959 (23.9%)	3236 (16.3%)	18,309 (24.7%)
Non-Hispanic Asian	1557 (4.6%)	1588 (1.7%)	983 (5.0%)	1381 (1.9%)
Other	2506 (7.4%)	7472 (7.8%)	1556 (7.8%)	6519 (8.8%)
**Additional Maternal Characteristics**				
Foreign-Born (outside of the U.S.)	12,437 (36.1%)	27,872 (28.7%)	7082 (35.0%)	20,058 (26.7%)
Married	13,260 (38.5%)	23,264 (24.0%)	7661 (37.9%)	16,057 (21.4%)
Education Less than High School^b^	3660 (10.4%)	11,414 (11.8%)	1778 (8.8%)	7533 (10.0%)
Late or No Entry to prenatal care (not within the first 3 months of pregnancy)	9746 (28.3%)	26,509 (27.3%)	5815 (28.7%)	20,671 (27.5%)

Abbreviations: g: grams; SD: Standard Deviation; WIC: Special Supplemental Nutrition Program for Women, Infants, and Children

^a^ This represents ALL Hispanic White ethnicity mothers (except Cuban)

^b^ For age > 20 years

**Fig. 1:**
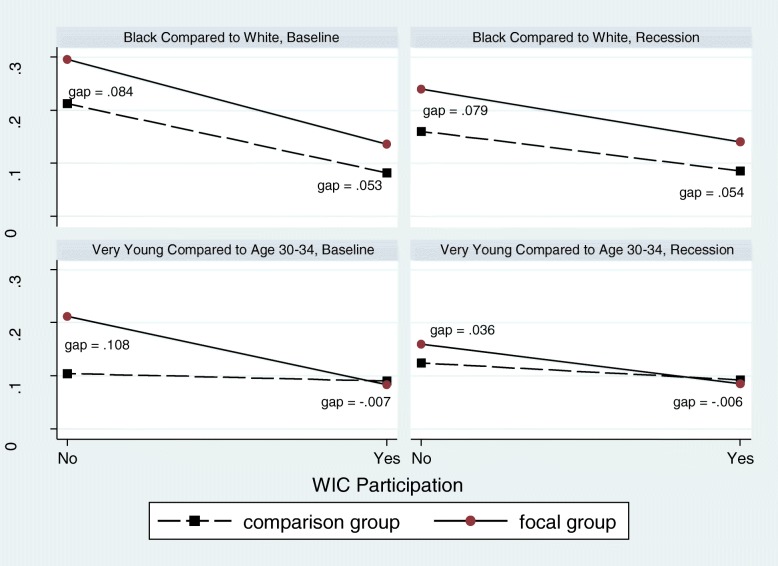
Reductions in the Probability of Low Birth Weight for Race Ethnicity and Maternal Age Factoring in WIC Interactions Before and During the Recession in Washington State and Florida (2005–2009)

Regression models to test for interactions between WIC and various socio-demographic characteristics identified significant interaction effects during both periods (baseline and Great Recession). WIC enrollment was associated with reductions in birth weight disparities for both Black women and those who entered prenatal care late or not at all compared to White women and those who entered prenatal care in the first trimester (Table 3). Specifically, WIC enrollment among Black mothers was associated with a reduction in the Black-White difference in the probability of delivering a LBW infant (Table 3, with supporting visual in Fig. 1). Black mothers without WIC, both at baseline and Recession, experienced greater probability of LBW compared to White women without WIC (0.084 (95% CI = 0.07, 0.10) vs. 0.080 (95% CI = 0.07, 0.09) respectively). This higher probability compared to White women was reduced for Black women with WIC by − 0.031 during baseline (95% CI = − 0.04, − 0.02) and − 0.025 in the Great Recession (95% CI = − 0.04, − 0.01). Compared to the infants of White mothers with WIC, infants of Black mothers using WIC had a gap of 0.053 baseline/0.054 Recession in probability of LBW controlling for all other factors.

**Table 3:** Differences in probability of low birth weight in relation to WIC and maternal characteristic interaction effects before and during the Great Recession using a linear probability regression model

	Baseline	Recession
	Coef. (95% CI)	Coef. (95% CI)
**Maternal WIC**		
Not Enrolled	Referent	Referent
Enrolled	0.005 (− 0.02, 0.03)	0.016 (− 0.00, 0.04)
**Maternal Race/Ethnicity**		
White, Non-Hispanic	Referent	Referent
White, Hispanic	0.019 (0.01, 0.03)	0.019 (0.01, 0.03)
Black, Non-Hispanic	0.084 (0.07, 0.10)	0.079 (0.07, 0.09)
Asian	0.034 (0.02, 0.05)	0.047 (0.02, 0.07)
Other	0.014 (0.00, 0.02)	0.010 (− 0.00, 0.02)
**WIC x Maternal Race/Ethnicity**^a^		
White x WIC	Referent	Referent
Hispanic x WIC	− 0.012 (− 0.02, 0.00)	− 0.010 (− 0.03, 0.01)
Black x WIC	− 0.031 (− 0.04, − 0.02)	−0.025 (− 0.04, − 0.01)
Asian x WIC	−0.014 (− 0.03, 0.01)	−0.022 (− 0.06, 0.01)
Other x WIC	− 0.010 (− 0.02, 0.00)	−0.007 (− 0.02, 0.01)
**Maternal Age (in years)**		
Age ≤ 14	0.108 (0.02, 0.19)	0.036 (−0.07, 0.14)
Age 15–19	0.002 (−0.02, 0.02)	− 0.002 (− 0.03, 0.02)
Age 20–24	−0.019 (− 0.03, − 0.01)	−0.016 (− 0.03, − 0.00)
Age 25–29	−0.017 (− 0.03, − 0.01)	−0.015 (− 0.03, 0.00)
Age 30–34	Referent	Referent
Age 35–39	0.020 (0.01, 0.04)	0.027 (−0.01, 0.06)
Age 40 +	0.068 (0.02, 0.12)	0.063 (0.03, 0.10)
**WIC x Maternal Age (in years)**		
Age ≤ 14 x WIC	−0.115 (− 0.20, − 0.03)	−0.042 (− 0.16, 0.08)
Age 15–19 x WIC	−0.027 (− 0.06, 0.00)	−0.011 (− 0.05, 0.02)
Age 20–24 x WIC	−0.007 (− 0.03, 0.01)	−0.010 (− 0.03, 0.01)
Age 25–29 x WIC	−0.002 (− 0.02, 0.02)	−0.000 (− 0.03, 0.03)
Age 30–34 x WIC	Referent	Referent
Age 35–39 x WIC	0.013 (− 0.01, 0.03)	−0.015 (− 0.06, 0.03)
Age 40 + x WIC	−0.016 (− 0.08, 0.04)	−0.014 (− 0.07, 0.04)
**Maternal Education**		
Less than H.S.	0.023 (0.01, 0.04)	0.040 (0.02, 0.06)
H.S. Diploma	0.017 (0.01, 0.03)	0.021 (0.01, 0.03)
Some College	Referent	Referent
Not Assessed; maternal age < 20 years	0.009 (−0.00, 0.02)	0.023 (0.00, 0.04)
**WIC x Maternal Education**		
Less than H.S. x WIC	−0.007 (− 0.02, 0.01)	−0.025 (− 0.05, − 0.00)
H.S. Diploma x WIC	−0.006 (− 0.01, 0.00)	−0.012 (− 0.03, 0.00)
Some College x WIC	Referent	Referent
Not Assessed; maternal age < 20 years x WIC	0.004 (−0.01, 0.02)	− 0.026 (− 0.03, − 0.01)
**Timing of Prenatal Care Entry**		
During First Trimester	Referent	Referent
After First Trimester (including no prenatal care)	0.005 (−0.00, 0.01)	0.017 (0.01, 0.03)
**WIC x Prenatal Care**		
First Trimester x WIC	Referent	Referent
Late x WIC	−0.012 (− 0.02, − 0.01)	−0.023 (− 0.03, − 0.01)

Abbreviations: CI: Confidence Interval; H.S.: high school; WIC: Special Supplemental Nutrition Program for Women, Infants, and Children

^a^ “x” represents interaction between variable (e.g. maternal race/ethnicity) and WIC

Women who entered prenatal care late without WIC enrollment had a greater probability of LBW outcomes of 0.005 (95% CI = − 0.00, 0.01) baseline and 0.017 (95% CI = 0.01, 0.03) at baseline and during the Great Recession period respectively, compared to those without WIC but with appropriate first trimester prenatal care. The interaction shows that late or no prenatal care individuals with WIC reduced this gap by − 0.012 (95% CI = − 0.02, − 0.01) baseline and − 0.023 (95% CI = − 0.03, − 0.01) during the Recession such that they were less likely to have LBW outcomes compared to women with timely prenatal care.

During the baseline period, WIC was also associated with a reduction in disparities for young mothers (age 14 years and under) compared with mothers who were age 30–34 years. Young mothers without WIC had a higher probability of LBW (baseline 0.108 95% CI = 0.02, 0.19) compared with the referent group (maternal age 30–34 years); with WIC, young women reduced this difference between the two age groups by − 0.115 (95% -0.20, − 0.03). During the Great Recession period, this age interaction with WIC was no longer significant for mothers age 14 years and under. However, in this period, education effects did have significant interactions with WIC. For those with less than a high school education and for those under age 20 years with their education unassessed, being enrolled in WIC reduced the difference observed for these categories compared to individuals with some college. (Note: Young age and finishing high school are difficult to disentangle; as a result, high school completion is only assessed for those over age 20 years).

**Fig. 2:**
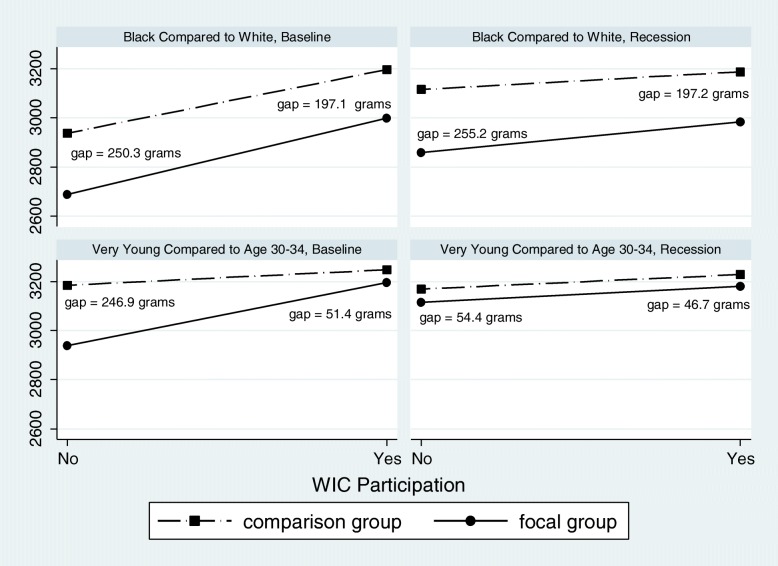
Reductions in the Gap in Birth Weight in Grams for Race/Ethnicity and Maternal Age Factoring in WIC Interactions Before and During the Great Recession in Washington State and Florida (2005–2009)

Figure 1 illustrates the results for probability of LBW for race and for age and illustrates the significant reductions in the observed gaps in outcomes due to WIC participation. (Similar results for birth weight in grams are in Fig. 2).

Table 4 presents the results of our regression models and the selected characteristics by WIC interactions for the continuous outcome variable—birth weight in grams.

**Table 4:** Linear regression model results showing differences due to WIC interactions for birth weight in grams and maternal characteristics before and during the Great Recession

	Baseline	Recession
	Coef. (95% CI)	Coef. (95% CI)
**Maternal WIC**		
Not Enrolled	Referent	Referent
Enrolled	3..0 (− 34.3, 40.2)	−17.5 (− 54.4, 19.3)
**Maternal Race/Ethnicity**		
White, Non-Hispanic	Referent	Referent
White, Hispanic	− 77.2 (− 103.8, − 50.6)	−76.5 (− 109.5, − 43.4)
Black, Non-Hispanic	−250.5 (− 276.1, − 224.9)	−255.2 (− 285.1, − 225.3)
Asian	−186.6 (− 216.3, − 152.8)	−208.1 (− 248.6, − 167.7)
Other	−38.6 (− 66.0, − 11.1)	−32.2 (− 62.4, − 1.9)
**WIC x Maternal Race/Ethnicity**^a^		
White x WIC	Referent	Referent
Hispanic x WIC	14.0 (− 18.0, 45.9)	27.5 (−4.5, 59.6)
Black x WIC	53.5 (32.4, 74.5)	58.03 (27.77, 88.30)
Asian x WIC	−3.4 (−43.6, 36.8)	44.7 (−4.6, 94.0)
Other x WIC	37.2 (18.9, 55.5)	38.5 (11.6, 65.3)
**Maternal Age (in years)**		
Age ≤ 14	−246.9 (− 376.2, − 117.7)	−54.4 (− 196.2, 87.4)
Age 15–19	− 28.9 (− 74.8, 17.1)	−24.4 (− 77.8, 23.1)
Age 20–24	28.7 (5.7, 51.6)	22.0 (−1.7, 45.6)
Age 25–29	32.1 (13.4, 50.7)	37.5 (7.8, 67.2)
Age 30–34	Referent	Referent
Age 35–39	− 50.8 (− 86.8, − 14.8)	− 45.5 (− 107.6, 16.6)
Age 40 +	− 169.7 (− 250.6, − 89.7)	−118.2 (− 179.7, − 56.7)
**WIC x Maternal Age (in years)**		
Age ≤ 14 x WIC	195.6 (81.1, 310.0)	7.7 (− 159.0, 174.4)
Age 15–19 x WIC	34.7(− 13.6, 83.0)	35.5 (−27.8, 98.8)
Age 20–24 x WIC	− 3.0 (− 31.9, 26.0)	5.3 (− 24.1, 34.6)
Age 25–29 x WIC	− 9.8 (− 34.2, 14.6)	−14.5 (− 57.8, 28.8)
Age 30–34 x WIC	Referent	Referent
Age 35–39 x WIC	− 34.8 (− 77.8, 8.2)	3.1 (− 74.6, 80.7)
Age 40 + x WIC	33.4 (− 50.6, 117.4)	− 10.9 (− 110.2, 88.3)
**Maternal Education**		
Less than H.S.	− 78.0 (− 115.0, − 41.1)	−86.6 (− 127.5, − 45.6)
H.S. Diploma	− 39.6 (− 58.3, − 20.8)	− 55.8 (− 77.4, − 34.3)
Some College	Referent	Referent
Not Assessed; maternal age < 20 years	− 39.9 (− 67.8, − 12.1)	−44.6 (− 75.7, − 13.6)
**WIC x Maternal Education**		
Less than H.S. x WIC	22.9 (−11.3, 57.0)	27.40 (− 12.76, 67.56)
H.S. Diploma x WIC	10.5 (− 7.7, 28.7)	26.02(− 1.69, 53.74)
Some College x WIC	Referent	Referent
Not Assessed; maternal age < 20 years x WIC	− 1.0 (− 34.8, 32.9)	16.9 (−20.3, 54.0)
**Timing of Prenatal Care Entry**		
During First Trimester	Referent	Referent
After First Trimester (including no prenatal care)	− 30.0 (− 5.1, − 15.0)	−39.6 (− 59.0, − 20.2)
**WIC x Prenatal Care**		
First Trimester x WIC	Referent	Referent
Late x WIC	37.0 (21.3, 52.6)	48.9 (26.5, 71.3)

Abbreviations: CI: Confidence Interval; H.S.: high school; WIC: Special Supplemental Nutrition Program for Women, Infants, and Children

^a^ “x” represents interaction between variable (e.g. maternal race/ethnicity) and WIC

Similarly to LBW, there were beneficial WIC interactions for infants of Black mothers and for those who entered prenatal care late or not at all during both periods. Among infants of Black mothers, WIC enrollment was associated with a decrease in the Black/White gap in birth weight. Specifically, among infants of Black mothers, WIC enrollment was associated with an increase in birth weight of 53.5 g (95% CI = 32.4, 74.5) (baseline) and 58.0 g (95% CI = 27.8, 88.3) (Recession), to 197.1 and to 197.2 less than White mothers using WIC during the same time periods. While these birth weights were still less than those of infants of White mothers, infants of Black mothers without WIC were 250.5 (95% CI = − 276.1, − 224.9) (baseline)/255.2 (95% CI = − 285.1, − 225.3) (Recession) grams less than comparable White mothers not enrolled in WIC.

Among mothers with late or no prenatal care, WIC interaction effects were associated with an additional 37.0 (95% CI = 21.3, 52.6) (baseline)/48.9 (95% CI = 26.5, 71.3) (Recession) grams which brought them up to 7.0 (baseline)/9.3 (Recession) grams higher than those infants of WIC participating women who entered prenatal care on time. During the baseline period, but not during the Great Recession, there was a positive WIC interaction among infants of young mothers (age 14 years and under). Those young mothers without WIC were associated with having infants 246.9 g (95% CI = − 376.2, − 117.7) less than their referent group (women age 30–34 years) while those young mothers with WIC were associated with having infants only 51.4 g less than their referent group (WIC enrolled women age 30–34 years). Additional tables [see Additional files 1 and 2] show complete results of both regression models.

## Discussion

This paper supports previous research linking WIC services to improved birth weights (both reducing LBW and increasing infant birth weight in grams) among some high-disadvantage groups [7–9]. It is encouraging that study results show that WIC has a fairly consistent effect for some key statuses (which did not appear to diminish over the course of the Great Recession). However, clear gaps are still present among other social and demographic characteristic groups (e.g., for unmarried mothers) for whom we did not find WIC to be associated with any detectable value in promoting better birth weight outcomes. Our takeaway from this is that WIC funders and providers should track and develop innovative approaches to address differential benefits among program participants. Future research needs to examine how WIC (and/or other maternal and child health programs) could be made to work better and reach farther to address persistent disparities in birth weight outcomes.

Consistent with previous studies, we found beneficial WIC interaction effects on birth weight between race and WIC, between prenatal care and WIC, and between age and WIC during the study period in Washington and Florida [7, 8, 15, 17]. For most groups, this beneficial relationship was stable before and during the Great Recession. However, the strength of the association among women who entered prenatal care later or not at all and had WIC nearly doubled from baseline to the Great Recession (in terms of reduced probability of LBW). The reduction due to WIC for infants of those women who entered prenatal care late and had WIC increased from baseline to the Great Recession; with the result that mothers who entered prenatal care late but enrolled in WIC had a just slightly lower chance of having a LBW baby than those who entered prenatal care on time and had WIC during either period other characteristics being equal. As such, it appears that the positive effects of WIC may have become more pronounced for this late/no prenatal group during the Great Recession. This may be due to WIC having more of an effect during a stressed/difficult time, or to changes in the population using WIC during the Great Recession (e.g., whether women with different characteristics became eligible and started using WIC).

Recent research has suggested that WIC recruitment efforts and program supports during the Great Recession were attenuated by cuts to local health department budgets/staffing [22, 23] —despite millions of dollars being unused for WIC response to increased need during the Great Recession [21]. Prah (2012) also reported that some potential recipients did not avail themselves of WIC services during the Great Recession—despite increased need—as they found the process of enrolling in the WIC program to be more troublesome than it was worth [40]. Prah cites the relative ease of enrolling in and using SNAP, as well as how much the benefit is worth compared to WIC, as one possible explanation for lower than expected increases in WIC enrollment during the Great Recession [40]. The average person on SNAP (food stamps) received $134/month and the average mother on WIC received $50/month in 2011. SNAP benefits were also accessed via unobtrusive debit cards while throughout the Great Recession most WIC sites continued to use paper vouchers [40]. While we were not able to include SNAP utilization data in this study, it will be valuable to see—as the WIC program shifts increasingly to electronic benefit methods—whether more individuals eligible for WIC services enroll and what the impact of these services will be on maternal and child health outcomes among WIC recipients [5].

### Limitations

While providing evidence for associations among birth weight, WIC, and other covariates, it is not possible to establish causal relationships with retrospective cross-sectional data, and findings must be interpreted with caution. Our study population may also limit generalizability of our results. For example, as described earlier we included only data from Washington and Florida. In this study we also limited inclusion to those with Medicaid or self-pay (uninsured) as the payer for their delivery, since we did not have maternal income or other specific information to be able to assess WIC eligibility. All Medicaid recipients are eligible for WIC services and, as found in other studies, it appears that WIC recipients were more likely to have disadvantaging characteristics (e.g., more likely to have less than a high school education, and/or less likely to be married), yet WIC was demonstrated to be particularly beneficial for some demographic and social groups [7]. Those who were self-pay (uninsured) were also included, as over 50% were WIC enrollees during each study year (confirming that they were a predominantly high-need group). It would be valuable to confirm these results in a broader WIC-eligible population—including among more race/ethnicity groups (e.g., Cuban women). Further, it was not possible with the data used in this study to identify when mothers enrolled in WIC and/or Medicaid, so it is possible that we did not capture differential dose/response relationships between having a larger benefit and being enrolled in WIC for a longer time—which may understate the magnitude of WIC effects on birth weight. We also did not assess the impacts of gestational age on birth weight in this cohort as our focus was on maternal characteristics that could be used to target WIC services for mothers. A future study could also include infant characteristics such as gestational age and/or weight/length in analyses. Finally, during the Great Recession there were massive increases in enrollments/payments for SNAP and unemployment insurance and we were not able to capture these services in our dataset to assess contributions to infant birth weight of other government supports beyond, instead of, or in addition to WIC.

## Conclusions

This paper adds to the evidence base linking WIC services to improved birth weight (both reducing low birth weight and increasing birth weight in grams) among some population groups. In particular, WIC appears to have been beneficial at decreasing disparity gaps in infant birth weight (reducing low birth weight; increasing birth weight in grams) among the very young, Black, and late/no prenatal care enrollees in this high-need population. It is encouraging that these benefits were present both before and during the Great Recession. However,clear gaps are still present among other social and demographic characteristic groups (e.g., for unmarried mothers) for which we did not find WIC to be associated with any detectable value in promoting better birth weight outcomes. Additional research into how and why WIC is more effective for some groups than others will be essential to further narrowing and eventually eliminating disparities. Finally, recent research has suggested that WIC recruitment efforts and program supports during the Great Recession were attenuated by cuts to local health department budgets/staffing. In preparation for future economic downturns it will be important to preserve and, if possible, expand WIC services during times of increased need.

## Supplementary information

**Additional file 1.** Word file, “Complete regression model results for low birth weight during baseline and Great Recession periods: Washington State and Florida, 2005-2009”.

**Additional file 2.** Word file, “Complete regression model results for birth weight in grams during baseline and Great Recession periods: Washington State and Florida, 2005-2009”.

## Data Availability

The data that support the findings of this study are available from the Washington State Department of Health and the Florida State Department of Health but restrictions apply to the availability of these data, which were used under license for the current study, and so are not publicly available. Data are however available from the authors upon reasonable request and with permission of the State Departments of Health in Washington and Florida.
