# Supplementary material for: The effect of women, infant, and children (WIC) services on birth weight before and during the 2007–2009 great recession in Washington state and Florida: a pooled cross-sectional time series analysis
Source: BMC Pregnancy Childbirth. 2020 Apr 28;20:252. doi: 10.1186/s12884-020-02937-5 (PMC7189643; doi:10.1186/s12884-020-02937-5)
Supplement: Supplementary file 1 — Additional file 1. Word file, “Complete regression model results for low birth weight during baseline and Great Recession periods: Washington State and Florida, 2005-2009”. [file 12884_2020_2937_MOESM1_ESM.docx]

**Table 5.** Complete regression model results for low birth weight during baseline /

Recession periods: Washington State and Florida, 2005-2009

|  | **Baseline** | | |  | | **Recession** | |  |
| --- | --- | --- | --- | --- | --- | --- | --- | --- |
|  | **Coef.**  **(Standard**  **Error)** | | | **95% CI** | | **Coef. (Standard Error )** | | **95% CI** |
| **Maternal Race/**  **Ethnicity** | | | | | | | | |
| White, Non-Hispanic (1) | Referent | | |  | | Referent | |  |
| White, Hispanic (2) | 0.018 (0.005) | | | 0.008 − 0.029 | | 0.019 (0.007) | | 0.005 − 0.032 |
| Black, Non-Hispanic (3) | 0.084 (0.005) | | | 0.073 − 0.095 | | 0.079 (0.007) | | 0.066 − 0.093 |
| Asian (4) | 0.034 (0.007) | | | 0.020 − 0.048 | | 0.047 (0.014) | | 0.019 − 0.074 |
| Other (5) | 0.014 (0.006) | | | 0.003 − 0.020 | | 0.010 (0.007) | | -0.003 − 0.024 |
| **WIC x Maternal**  **Race/Ethnicity** | | | | | | | | |
| White x WIC | Referent | | |  | | Referent | |  |
| Hispanic x WIC | -0.012 (0.006) | | | -0.024 − 0.000 | | -0.010 (0.008) | | -0.025 − 0.005 |
| Black x WIC | -0.031 (0.006) | | | -0.042 − -0.019 | | -0.025 (0.007) | | -0.039 − -0.011 |
| Asian x WIC | -0.014 (0.010) | | | -0.033 − 0.005 | | -0.022 (0.017) | | -0.056 − 0.011 |
| Other x WIC | -0.010 (0.006) | | | -0.022 − 0.001 | | -0.007 (0.007) | | -0.021 − -0.007 |
| **Maternal Age** | | | | | | | | |
| Age < or = 14 (1) | 0.108 (0.043) | | | 0.023 − 0.193 | | 0.036 (0.053) | | -0.069 − 0.141 |
| Age 15-19 (2) | 0.002 (0.010) | | | -0.018 − 0.021 | | -0.002 (0.014) | | -0.029 − 0.024 |
| Age 20-24 (3) | -0.019 (0.006) | | | -0.031 − -0.006 | | -0.016 (0.006) | | -0.028 − -0.004 |
| Age 25-29 (4) | -0.017 (0.006) | | | -0.029 − -0.006 | | -0.015 (0.009) | | -0.033 − 0.004 |
| Age 30-34 (5) | Referent | | |  | | Referent | |  |
| Age 35-39 (6) | 0.020 (0.008) | | | 0.005 − 0.035 | | 0.027 (0.017) | | -0.007 − 0.061 |
| Age 40 + (7) | 0.068 (0.024) | | | 0.021 − 0.115 | | 0.063 (0.016) | | 0.032 − 0.095 |
| **Maternal Age x**  **WIC** | | | | | | | | |
| Age < or = 14 x WIC | -0.116 (0.043) | | | -0.201 − -0.030 | | -0.043 (0.059) | | -0.160 − 0.075 |
| Age 15-19 x WIC | -0.027 (0.014) | | | -0.056 − 0.001 | | -0.011 (0.017) | | -0.045 − 0.023 |
| Age 20-24 x WIC | -0.007 (0.010) | | | -0.027 − 0.013 | | -0.010 (0.009) | | -0.027 − 0.007 |
| Age 25-29 x WIC | -0.002 (0.009) | | | -0.020 − 0.016 | | -0.000 (0.013) | | -0.026 − 0.025 |
| **Maternal Age x WIC (cont.)** |  | | |  | |  | |  |
| Age 30-34 x WIC | Referent | | |  | | Referent | |  |
| Age 35-39 x WIC | 0.013 (0.009) | | | -0.005 − 0.031 | | -0.015 (0.020) | | -0.055 − 0.025 |
| Age 40 + x WIC | -0.016 (0.030) | | | -0.076 − 0.044 | | -0.014 (0.029) | | -0.071 − 0.043 |
| **Marital Status** | | | | | | | | |
| Married | | Referent | |  | | Referent | |  |
| Unmarried | | 0.015 (0.004) | | 0.007 − 0.022 | | 0.011 (0.004) | | 0.003 − 0.018 |
| **Marital Status x**  **WIC** | | | | | | | | |
| Married x WIC | | Referent | |  | | Referent | |  |
| Unmarried x WIC | | -0.008 (0.005) | | -0.017 − 0.001 | | -0.003 (0.004) | | -0.011 − 0.005 |
| **Maternal Birthplace** | | | | | | | | |
| Born in US | | Referent | |  | | Referent | |  |
| Born outside US | | -0.021 (0.006) | | -0.033 − -0.009 | | -0.022 (0.007) | | -0.036 − -0.009 |
| **Maternal Birthplace**  **x WIC** | | | | | | | | |
| Born in US x WIC | | Referent | |  | | Referent | |  |
| Born Outside US x WIC | | 0.006 (0.005) | | -0.003 − 0.015 | | 0.008 (0.006) | | -0.003 − 0.020 |
| **Maternal Education** | | | | | | | | |
| Less than H.S. (1) | | 0.023 (0.006) | | 0.012 − 0.035 | | 0.040 (0.010) | | 0.019 − 0.060 |
| H.S. Diploma (2) | | 0.017 (0.004) | | 0.008 − 0.025 | | 0.021 (0.005) | | 0.011 − 0.032 |
| Some College (3) | | Referent | |  | | Referent | |  |
| Not Assessed; maternal age < 20 (4) | | 0.023 (0.006) | | -0.003 − 0.021 | | 0.023 (0.009) | | 0.004 − 0.041 |
| **Maternal Education**  **X WIC** | | | | | | | | |
| Less than H.S. x WIC | | -0.007 (0.006) | | -0.019 − 0.005 | | -0.025 (0.012) | | -0.048 − -0.002 |
| H.S. Diploma x WIC | | -0.006 (0.004) | | -0.014 − 0.003 | | -0.013 (0.007) | | -0.025 − 0.000 |
| Some College x WIC | | Referent | |  | | Referent | |  |
| Not Assessed; maternal age < 20 x WIC | | 0.004 (0.009) | | -0.013 − 0.021 | | -0.026 (0.010) | | -0.047 − -0.005 |
| **Timing of Prenatal**  **Care Entry** | |  | |  | |  | |  |
| During First Trimester | | Referent | |  | | Referent | |  |
| After First Trimester (including no prenatal care) (1) (cont.) | | 0.005 (0.004) | | -0.003 − 0.013 | | 0.017 (0.005) | | 0.008 − 0.027 |
| **Prenatal Care X**  **WIC** | | | | | | | | |
| First Trimester x WIC | | Referent | |  | | Referent | |  |
| Late x WIC | | -0.012 (0.004) | | -0.020 − -0.005 | | -0.023 (0.005) | | -0.034 − -0.013 |
| **Maternal WIC** | | | | | | | | |
| Not Enrolled | | Referent | |  | | Referent | |  |
| Enrolled (1) | | 0.005 (0.012) | | -0.019 − 0.029 | | 0.016 (0.010) | | -0.004 − 0.035 |
| **Medicaid or**  **Uninsured** | | | | | | | | |
| Medicaid | | Referent |  | | Referent | |  | |
| Uninsured (3) | | -0.009  (0.003) | -0.016 − -0.003 | | -0.008 (0.006) | | -0.019 − 0.003 | |
| **Medicaid or**  **Uninsured X WIC** | | | | | | | | |
| Medicaid x WIC | | Referent |  | | Referent | |  | |
| Uninsured x WIC | | -0.002 (0.007) | -0.015 − 0.012 | | 0.002 (0.006) | | -0.010 − 0.013 | |
| **Geography** | | | | | | | | |
| Metropolitan (1) | | Referent |  | | Referent | |  | |
| Micropolitan (2) | | - 0.019 (0.006) | -0.032 − -0.007 | | 0.006 (0.013) | | -0.019 − 0.031 | |
| Rural (3) | | -0.017 (0.011) | -0.039 − 0.005 | | 0.017 (0.014) | | -0.012 − 0.045 | |
| **Geography X WIC** | | | | | | | | |
| Metropolitan x WIC | | Referent |  | | Referent | |  | |
| Micropolitan x WIC | | -0.000 (0.008) | -0.015 − 0.015 | | -0.010 (0.013) | | -0.036 − 0.016 | |
| Rural x WIC | | 0.008 (0.010) | -0.012 − 0.028 | | -0.017 (0.015) | | -0.048 − 0.013 | |
| **Community Poverty** | | | | | | | | |
| Residence in Top 1/3 Poorest Local Health Jurisdictions | | 0.009  (0.004) | 0.002 − 0.017 | | 0.008 (0.004) | | -0.001 − 0.016 | |
| Residence in Other 2/3 of Local Health Jurisdictions (non-poor) | | Referent |  | | Referent | |  | |
| **State** | | | | | | | | |
| Florida (1) | | Referent |  | | Referent | |  | |
| Washington (2) | | -0.006 (0.005) | -0.015 − 0.003 | | -0.014 (0.004) | | -0.023 − -0.006 | |
| **Unemployment Rate** | | 0.000 (0.002) | -0.003 − 0.003 | | -0.000 (0.000) | | -0.001 − 0.001 | |
| **Median Household Income** | | -0.000  (0.000) | -0.000 − -0.000 | | -0.000 (0.000) | | -0.000 − 0.000 | |
| **Gini Coefficient** | | 0.047 (0.040) | -0.033 − 0.127 | | 0.001 (0.030) | | -0.061 − 0.060 | |
| **Percent Voting Republican in 2004 or 2008 Presidential Election** | | 0.0003  (0.0001) | 0.00008 − 0.0006 | | -0.0000 (0.0001) | | -0.0002 − 0.0002 | |
| **Per Capital FM and GP Physicians** | | -0.209  (0.091) | -0.389 − -0.029 | | 0.063 (0.095) | | -0.125 − 0.252 | |
| **Per Capita Local**  **Health Department**  **Expenditures** | | | | | | | | |
| 2maternal and child health (lagged) | | 0.0003  (0.0001) | 0.0001 − 0.0006 | | -0.0002  (0.0002) | | -0.0005 − 0.0001 | |
| WIC (lagged) | | 0.0005  (0.0004) | -0.0003 − 0.0012 | | 0.0015  (0.0005) | | 0.001 − 0.002 | |
| Constant | | 0.060  (0.028) | 0.005 − 0.116 | | 0.069 (0.024) | | 0.021 -0.117 | |

Abbreviations: CI: Confidence Interval; FM: family medicine; GP: general practitioner; H.S.: high school; US: United States; WIC: Special Supplemental Nutrition Program for Women, Infants, and Children.
