# Supplementary material for: The effect of women, infant, and children (WIC) services on birth weight before and during the 2007–2009 great recession in Washington state and Florida: a pooled cross-sectional time series analysis
Source: BMC Pregnancy Childbirth. 2020 Apr 28;20:252. doi: 10.1186/s12884-020-02937-5 (PMC7189643; doi:10.1186/s12884-020-02937-5)
Supplement: Supplementary file 2 — Additional file 2. Word file, “Complete regression model results for birth weight in grams during baseline and Great Recession periods: Washington State and Florida, 2005-2009”. [file 12884_2020_2937_MOESM2_ESM.docx]

**Table 6.** Complete regression model results for birth weight in grams during baseline and

Recession periods: Washington State and Florida, 2005-2009

|  | | **Baseline** | | | |  | | **Recession** | | | |  |
| --- | --- | --- | --- | --- | --- | --- | --- | --- | --- | --- | --- | --- |
|  | | **Coef. (Standard Error)** | | | | **95% CI** | | **Coef.**  **(Standard Error )** | | | | **95% CI** |
| **Maternal Race/**  **Ethnicity** | | | | | | | | | | | | |
| White, Non-Hispanic (1) | | Referent | | | |  | | Referent | | | |  |
| White, Hispanic (2) | | -77.20 (13.43) | | | | -103.84 − -50.57 | | -76.45 (16.67) | | | | -109.52 − -43.39 |
| Black, Non-Hispanic (3) | | -250.53 (12.91) | | | | -276.13 − -224.92 | | -255.24 (15.07) | | | | -285.14 − -225.34 |
| Asian (4) | | -184.55 (16.02) | | | | -216.33 − -152.77 | | -208.12 (20.39) | | | | -248.56 − -167.68 |
| Other (5) | | -38.58 (13.84) | | | | -66.02 − -11.13 | | -32.17.18 (15.25) | | | | -62.42 − -1.92 |
| **WIC x Maternal**  **Race/Ethnicity** | | | | | | | | | | | | |
| White x WIC | | Referent | | | |  | | Referent | | | |  |
| Hispanic x WIC | | 13.96 (16.12) | | | | -18.01 – 45.93 | | 27.52 (16.14) | | | | -4.50 – 59.54 |
| Black x WIC | | 53.47 (10.62) | | | | 32.41 − 74.53 | | 58.03 (15.26) | | | | 27.77 − 88.29 |
| Asian x WIC | | -3.40 (20.28) | | | | -43.63 – 36.84 | | 44.69 (24.83) | | | | -4.57 – 93.95 |
| Other x WIC | | 37.18 (9.24) | | | | 18.86 – 55.49 | | 38.47 (13.53) | | | | 11.63 – 65.31 |
| **Maternal Age** | | | | | | | | | | | | |
| Age < or = 14 (1) | | -246.93 (65.17) | | | | -376.20 − -117.66 | | -54.41 (71.46) | | | | -196.18 – 87.36 |
| Age 15-19 (2) | | -28.86 (23.14) | | | | -74.77 – 17.05 | | -24.36 (25.41) | | | | -77.77 – 23.05 |
| Age 20-24 (3) | | 28.66 (11.56) | | | | 5.73 – 51.59 | | 21.96 (11.90) | | | | -1.66 – 45.57 |
| Age 25-29 (4) | | 32.06 (9.40) | | | | 13.42 − 50.70 | | 38.49 (14.96) | | | | 7.81 – 67.17 |
| Age 30-34 (5) | | Referent | | | |  | | Referent | | | |  |
| Age 35-39 (6) | | -50.81 (18.13) | | | | -86.77 − -14.84 | | -45.48 (31.31) | | | | -107.60 – 16.64 |
| Age 40 + (7) | | -169.66 (40.80) | | | | -250.60 − - 88.72 | | -118.16 (31.01) | | | | -179.67 − -56.65 |
| **Maternal Age**  **x WIC** | |  | | | |  | |  | | | |  |
| Age < or = 14 x WIC | | 195.55 (57.71) | | | | 81.06 – 310.03 | | 7.72 (84.04) | | | | -158.99 – 174.44 |
| Age 15-19 x WIC | | 34.72(24.35) | | | | -13.59 – 83.03 | | 35.49 (31.92) | | | | -27.83 − 98.82 |
| Age 20-24 x WIC | | -2.97 (14.59) | | | | -31.90 – 25.96 | | 5.26 (14.79) | | | | -24.08 − 34.60 |
| Age 25-29 x WIC | | -9.81 (12.30) | | | | -34.21 – 14.60 | | -14.49 (21.83) | | | | -57.80 – 28.82 |
| Age 30-34 x WIC | | Referent | | | |  | | Referent | | | |  |
| Age 35-39 x WIC | | -34.84 (21.68) | | | | -77.84 – 8.16 | | 3.07 (39.13) | | | | -74.55 – 80.69 |
| Age 40 + x WIC | | 33.40 (42.33) | | | | -50.57 − 117.36 | | -10.94 (50.02) | | | | -110.17 – 88.29 |
| **Marital Status** | | | | | | | | | | | | |
| Married | | Referent | | | |  | | Referent | | | |  |
| Unmarried | | -44.39 (9.79) | | | | -63.81 − -24.97 | | -35.87 (13.01) | | | | -61.67 − -10.07 |
| **Marital Status x WIC** | | | | | | | | | | | | |
| Married x WIC | | Referent | | | |  | | Referent | | | |  |
| Unmarried x WIC | | 14.73 (9.83) | | | | -4.76 – 34.22 | | 8.37 (14.75) | | | | -20.89 – 37.64 |
| **Maternal**  **Birthplace** | | | | | | | | | | | | |
| Born in US | | Referent | | | |  | | Referent | | | |  |
| Born outside US | | 22.44 (12.69) | | | | -2.73 – 47.60 | | 25.87 (14.97) | | | | -3.83 – 55.57 |
| **Maternal**  **Birthplace x WIC** | | | | | | | | | | | | |
| Born in US x WIC | | Referent | | | |  | | Referent | | | |  |
| Born Outside US x WIC | | 11.37 (11.15) | | | | -10.76 – 33.49 | | 4.39 (13.72) | | | | -22.82 – 31.60 |
| **Maternal**  **Education** | |  | | | |  | |  | | | |  |
| Less than H.S. (1) | | -78.02 (18.62) | | | | -114.96 − -41.09 | | -86.57 (20.63) | | | | -127.50 − -45.64 |
| H.S. Diploma (2) | | -39.59 (9.45) | | | | -58.34 − -20.84 | | -55.84 (10.85) | | | | -77.36 − -34.31 |
| Some College (3) | | Referent | | | |  | | Referent | | | |  |
| Not Assessed; maternal age < 20 (4) | | -39.92 (14.03) | | | | -67.75 − -12.09 | | -44.62 (15.65) | | | | -75.66 − -13.59 |
| **Maternal**  **Education**  **x WIC** | | | | | | | | | | | | |
| Less than H.S. x WIC | | 22.88 (17.21) | | | | -11.27 − 57.03 | | 27.40 (20.25) | | | | -12.76 – 67.56 |
| H.S. Diploma x WIC | | 10.51 (9.18) | | | | - 7.70 − 28.72 | | 26.02 (13.97) | | | | -1.69 – 57.74 |
| Some College x WIC | | Referent | | | |  | | Referent | | | |  |
| Not Assessed; maternal age < 20 x WIC | | -0.96 (17.07) | | | | -34.83 – 32.90 | | 16.85 (18.73) | | | | -20.30 – 54.00 |
| **Timing of**  **Prenatal**  **Care Entry** | | | | | | | | | | | | |
| During First Trimester | Referent | | | | |  | | Referent | |  | | |
| After First Trimester (including no prenatal care) (1) | -30.00 (7.59) | | | | | -45.05 − -14.96 | | -39.61 (9.79) | | -59.03 − -20.18 | | |
| **Prenatal Care**  **x WIC** | | | | | | | | | | | | |
| \| First Trimester x WIC \| Referent \|  \| Referent \| \|  \| \| \| --- \| --- \| --- \| --- \| --- \| --- \| --- \| \| Late x WIC \| 36.97 (8.14) \| 20.03 − 52.33 \| \| 48.91 (11.30) \| \| 26.48 – 71.33 \| \| | | | | | | | | | | | | |
| **Maternal WIC** | | | | | | | | | | | | |
| Not Enrolled | Referent | | | | | |  | | Referent | |  | |
| Enrolled (1) | 2.95 (18.75) | | | | | | -34.25 – 40.15 | | -17.54 (18.58) | | -54.40 – 19.33 | |
| **Medicaid or**  **Uninsured** | | | | | | | | | | | | |
| Medicaid | | | Referent | |  | | | | Referent | |  | |
| Uninsured (3) | | | 33.75 (9.34) | | 15.23 – 52.28 | | | | 21.44 (13.06) | | -4.47 – 47.35 | |
| **Medicaid or**  **Uninsured x WIC** | | |  | |  | | | |  | |  | |
| Medicaid x WIC | | | Referent | |  | | | | Referent | |  | |
| Uninsured x WIC | | | -9.16 (11.74) | | -32.45 − 14.13 | | | | -1.57 (14.00) | | -29.34 – 26.20 | |
| **Geography** | | | | | | | | | | | | |
| Metropolitan (1) | | | Referent | |  | | | | Referent | |  | |
| Micropolitan (2) | | | 45.82 (18.93) | | 8.26 – 83.37 | | | | 70.97 (26.46) | | 18.48 − 123.46 | |
| Rural (3) | | | 43.79 (29.65) | | -15.03 – 102.61 | | | | 22.92 (27.72) | | -32.06 – 77.90 | |
| **Geography X**  **WIC** | | | | | | | | | | | | |
| Metropolitan x WIC | | | Referent | |  | | | | Referent | |  | |
| Micropolitan x WIC | | | 0.72 (21.55) | | -42.03 – 43.47 | | | | -45.99 (28.83) | | -103.18 – 11.21 | |
| Rural x WIC | | | -32.89 (29.96) | | -92.33 – 26.55 | | | | -20.38 (31.28) | | -82.43 – 41.66 | |
| **Community**  **Poverty** | | | |  | | |  |  | | |  | |
| Residence in Top 1/3 Poorest Local Health Jurisdictions | | -48.67 (11.42) | | | | | -71.33 − -26.01 | -31.53 (14.01) | | | -59.33 − -3.73 | |
| **State** | |  | | | | |  |  | | |  | |
| Florida (1) | | Referent | | | | |  | Referent | | |  | |
| Washington (2) | | 66.19 (15.43) | | | | | 35.59 – 96.79 | 62.66 (11.17) | | | 40.50 – 84.82 | |
| Florida (1) | | Referent | | | | |  | Referent | | |  | |
| **Unemployment Rate** | | 0.05 (3.89) | | | | | -7.67 – 7.76 | 1.71 (0.71) | | | 0.30 − 3.12 | |
| **Median Household Income** | | 0.00 (0.00) | | | | | -0.00 − 0.00 | 0.00 (0.00) | | | -0.00 − 0.00 | |
| **Gini Coefficient** | | -127.65 (113.58) | | | | | -352.96 – 97.66 | -137.45 (102.11) | | | -340.01 – 65.10 | |
| **Percent Voting Republican in 2004 or 2008 Presidential Election** | | -0.49 (0.26) | | | | | -1.02 − 0.03 | -0.07 (0.31) | | | -0.67 − 0.54 | |
| **Per Capital FM and GP Physicians** | | 479.93 (249.51) | | | | | -15.03 – 974.89 | 288.55 (270.09) | | | -247.23 – 824.34 | |
| **Per Capita**  **Local Health**  **Department**  **Expenditures** | | | | | | | | | | | | |
| 2maternal and child health (lagged) | | -0.04 (0.37) | | | | | -0.76 − 0.69 | 0.79 (0.41) | | | 0.03 − 1.60 | |
| WIC (lagged) | | -1.30 (0.99) | | | | | -3.27 − 0.67 | -3.33 (1.10) | | | -5.51 − -1.15 | |
| Constant | | 3326.13 (71.59) | | | | | 3184.12 − 3468.15 | 3328.89 (68.55) | | | 3192.90 – 3464.87.89 | |

Abbreviations: CI: Confidence Interval; FM: family medicine; GP: general practitioner; H.S.: high school; US: United States; WIC: Special Supplemental Nutrition Program for Women, Infants, and Children.
